# A NOVEL POSTDISCHARGE TELEHEALTH INTERVENTION FOR STROKE CAREGIVERS: IMPLICATIONS FOR IMPLEMENTATION

**DOI:** 10.1093/geroni/igad104.0559

**Published:** 2023-12-21

**Authors:** Jennifer LeLaurin, Nathaniel Eliazar-Macke, I Magaly Freytes, Constance Uphold

**Affiliations:** University of Florida College of Medicine, Gainesville, Florida, United States; U.S. Department of Veterans Affairs, Gainesville, Florida, United States; NF/SG VHS, Gainesville, Florida, United States; North Florida/South Georgia Veterans Health System, Gainesville, Florida, United States

## Abstract

This study investigated the potential for implementation of the RESCUE stroke caregiver telehealth intervention by assessing acceptability, feasibility, and cost. Family caregivers (n=174) of Veteran stroke survivors from eight medical centers were randomized to usual care or intervention. The RN-led intervention involved discharge plan review, problem-solving training, education, and support delivered via one telephone session and eight asynchronous online messaging center sessions. Acceptability was assessed using an adapted Acceptability and Enactment Survey (n=42) and qualitative interviews with a subsample of caregivers (n=16). We used retention data to assess feasibility and calculated cost per participant. Findings indicate the intervention was acceptable to caregivers. Most intervention completers found the intervention very/extremely helpful (90.5%), very/extremely easy to use (78.4%), and resolved most/all caregiving problems (64.3%). Qualitative interviews showed caregivers valued the asynchronous delivery method, personalized nature of the intervention, and ability of the RN to answer medical questions and provide education. More intervention caregivers dropped out compared to usual care (50.0% vs.19.3%, p< 0.001). Intervention dropout was predicted by lower education (p=0.03) and less positive views of caregiving (p=0.01). Cost was $309.98 per intervention completer. Findings support intervention acceptability and highlight the value of RN-led interventions. The relatively low cost and fully remote nature of the intervention support its suitability for widespread implementation. Multiple delivery options and flexible intervention schedules may enhance engagement. The RESCUE intervention provides a model of a low-cost post-discharge telehealth intervention for stroke caregivers that can be adapted for other conditions. Future work should test methods to augment retention.

